# *Helicobacter pylori* downregulates expression of human β-defensin 1 in the gastric mucosa in a type IV secretion-dependent fashion

**DOI:** 10.1111/cmi.12174

**Published:** 2013-08-07

**Authors:** SR Patel, K Smith, DP Letley, KW Cook, AA Memon, RJM Ingram, E Staples, S Backert, AM Zaitoun, JC Atherton, K Robinson

**Affiliations:** 1 Nottingham Digestive Diseases Biomedical Research Unit, University of NottinghamNottingham, NG7 2RD, UK; 2 Centre for Biomolecular Sciences, University of NottinghamNottingham, NG7 2RD, UK; 3 Lehrstuhl für Mikrobiologie, Department für Biologie, Friedrich-Alexander-Universität Erlangen Nürnberg91058, Erlangen, Germany; 4 Department of Cellular Pathology, Nottingham University Hospitals NHS TrustNottingham, NG7 2UH, UK

## Abstract

*Helicobacter pylori* establishes a chronic lifelong infection in the human gastric mucosa, which may lead to peptic ulcer disease or gastric adenocarcinoma. The human beta-defensins (hβDs) are antimicrobial peptides, hβD1 being constitutively expressed in the human stomach. We hypothesized that *H. pylori* may persist, in part, by downregulating gastric hβD1 expression. We measured hβD1 and hβD2 expression *in vivo* in relation to the presence, density and severity of *H. pylori* infection, investigated differential effects of *H. pylori* virulence factors, and studied underlying signalling mechanisms *in vitro*. Significantly lower hβD1 and higher hβD2 mRNA and protein concentrations were present in gastric biopsies from infected patients. Those patients with higher-level bacterial colonization and inflammation had significantly lower hβD1 expression, but there were no differences in hβD2. *H. pylori* infection of human gastric epithelial cell lines also downregulated hβD1. Using wild-type strains and isogenic mutants, we showed that a functional*cag* pathogenicity island-encoded type IV secretion system induced this downregulation. Treatment with chemical inhibitors or siRNA revealed that *H. pylori* usurped NF-κB signalling to modulate hβD1 expression. These data indicate that *H. pylori* downregulates hβD1 expression via NF-κB signalling, and suggest that this may promote bacterial survival and persistence in the gastric niche.

## Introduction

*Helicobacter pylori* persistently infects the stomachs of almost half the world's population. Although the majority of infected people remain asymptomatic, approximately 10–15% go on to develop peptic ulcer disease or gastric cancers. The disease outcome of an infection is determined by a combination of bacterial, host and environmental factors (Blaser and Atherton, 2004; Robinson *et al*., 2007; Atherton and Blaser, 2009). *H. pylori* expresses numerous virulence determinants that have been linked to disease, including the polymorphic vacuolating cytotoxin gene A (*vacA*) and the *cag* pathogenicity island (*cag*PAI) (Backert *et al*., 2010). *H. pylori* strains possessing toxic alleles of *vacA* manipulate epithelial and immune cell functions that contribute to disease. The *cag*PAI encodes a type IV secretion system (T4SS) that binds α_5_β_1_ integrin on host cells, penetrates and delivers the bacterial effector protein CagA (Odenbreit *et al*., 2000; Kwok *et al*., 2007). Once translocated into the cytosol, CagA activates specific signalling pathways, including MAP kinase and NF-κB-induced signalling. Both NF-κB p50/p65 heterodimers and p65 or p50 homodimers undergo nuclear translocation (Keates *et al*., 1997; Wada *et al*., 2001; Saha *et al*., 2008). This leads to the expression of a variety of pro-inflammatory and immune defence genes. The *cag*PAI also allows translocation of soluble bacterial cell wall components into the epithelial cytosol. These short-chain peptidoglycan derivatives (disaccharide tripeptides) are generated via activity of the lytic transglycosylase encoded by *slt* (HP0645), an enzyme normally involved in peptidoglycan remodelling. The disaccharide tripeptides are recognized by nucleotide-binding oligomerization domain 1 (NOD1), an intracellular sensor of Gram-negative bacteria, leading also to NF-κB-induced pro-inflammatory signalling (Viala *et al*., 2004; Brandt *et al*., 2005; Boughan *et al*., 2006). A third *cag*PAI-mediated pathway has recently been described, where interaction of CagL with the α_5_β_1_ integrin on epithelial cells also triggers MAP kinase and NF-κB activation (Gorrell *et al*., 2013). Bacterial factors therefore manipulate the gastric inflammatory response, which underlies the development of PUD and gastric cancer.

Antimicrobial peptides (AMPs) are important in the host response to infection. These small, cationic peptides are expressed by a number of cell types including epithelial cells. They can be subdivided into several categories, all of which are potent and cytotoxic against bacteria but not against normal mammalian cells (Guani-Guerra *et al*., 2010). One group, the human β-defensins (hβDs), is a crucial component of the host defence at mucosal epithelia (Zasloff, 2002; O'Neil, 2003). Expression of hβD2 and hβD4 is upregulated during *H. pylori* infection in a *cag*PAI-dependent and NF-κB-mediated manner, and these AMPs are known to have antimicrobial activity against the bacterium (George *et al*., 2003; Boughan *et al*., 2006; Hornsby *et al*., 2008; Otte *et al*., 2009). hβD3 also has bactericidal activity against *H. pylori* and its expression is initially upregulated by *H. pylori* infection *in vitro* (Boughan *et al*., 2006), but subsequently downregulated in a CagA-dependent manner during prolonged infection (Bauer *et al*., 2012).

hβD1 (encoded by *DEFB1*) is constitutively expressed in uninflamed normal tissue (Liu *et al*., 1997; O'Neil *et al*., 2000), which highlights its importance in protection against microbial infection. Expression in the GI tract (including the gastric mucosa) is predominantly by epithelial cells rather than inflammatory cells (Frye *et al*., 2000). One study found increased hβD1 expression in the *H. pylori* infected human gastric mucosa (Bajaj-Elliott *et al*., 2002), but a second found decreased expression (Taha *et al*., 2005). In a more recent study, a non-significant trend towards reduced levels of hβD1 mRNA was found in gastric biopsies from infected patients (Vordenbaumen *et al*., 2010). These studies, although somewhat contradictory, suggest that *H. pylori* may modulate hβD1 expression. Consistent with this idea are the observed binding motifs for multiple transcription factors, including NF-κB, in the promoter sequence of the *DEFB1* gene (Liu *et al*., 1997; Zhu *et al*., 2003; Prado-Montes de Oca *et al*., 2009).

Many AMPs also have chemotactic activity, working together to direct immune effector cells to the site of infection. Importantly, hβD1, hβD2 and hβD3 are associated with recruiting immature dendritic cells and memory T cells via CC-chemokine receptor 6 (CCR6), hence representing a bridge between the innate and adaptive immune responses (Yang *et al*., 2002). Cathelicidins have been found to be involved in the recruitment of neutrophils, in addition to circulating and tissue-derived monocytes (De *et al*., 2000). AMPs therefore act to induce pro-inflammatory immune responses, in some cases inducing immune mediators that further induce the expression of these AMPs, effectively creating a positive feedback loop (Zasloff, 2007). Therefore, downregulation of hβD1 could also mediate persistence of *H. pylori* infection by modulating the immune response.

The role of hβD1 during *H. pylori* infection is unclear and modulation of hβD1 expression by both host and bacterial factors may be possible. In this study, we therefore aimed to assess hβD1 expression levels in the *H. pylori* infected gastric mucosa in comparison with hβD2, to characterize the influence of *H. pylori* virulence determinants on hβD1 expression, and to determine the signalling pathways involved in regulating expression of this defensin during infection.

## Results

### *H. pylori* infection is associated with reduced hβD1 expression in the human stomach *in vivo*

**Figure 1:**
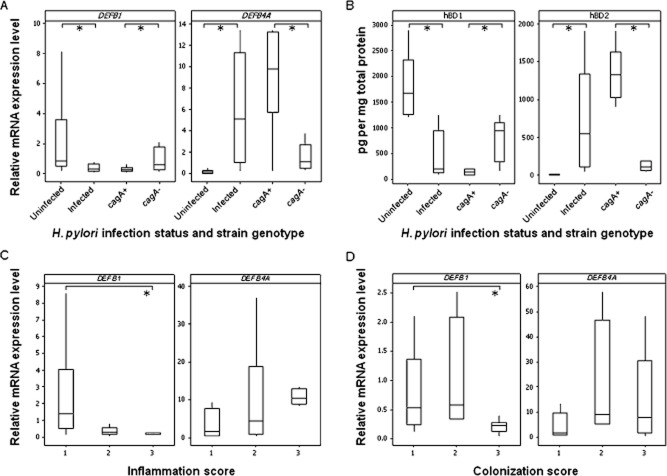
Analysis of hβD1 and hβD2 expression during *H. pylori* infection *in vivo*. Levels of *DEFB1* and *DEFB4A* mRNA were measured in the gastric mucosa of 23 uninfected and 31 *H. pylori* infected donors (A: *P = 0.005 and *P* = 0.001 respectively), and compared according to *cagA* genotype status of the colonizing strain (A: *P = 0.028 and *P* = 0.006 respectively). hβD1 and hβD2 protein concentrations in gastric biopsies from five uninfected and 10 *H. pylori* infected donors were measured (B: *P = 0.001 and *P* = 0.001), and concentrations in five *cagA*+ and five *cagA−* biopsies were also compared (B: *P = 0.016 and *P* = 0.004). Expression levels were also stratified based on histological inflammation scores graded from gastric biopsy tissue sections as mild (score of 1, *n* = 6), moderate (score of 2, *n* = 20) or substantial (score of 3, *n* = 5) (C: *P = 0.045). Data were also stratified according to bacterial density scores: mild (score of 1, *n* = 13), moderate (score of 2, *n* = 5) or substantial colonization (score of 3, *n* = 13) (D: *P = 0.001). RT-qPCR data were normalized against *GAPDH* and expressed relative to measurements from an uninfected tissue comparator. Protein concentrations were calculated per mg of total protein. Boxes represent the first and third quartiles with median values shown as a horizontal line within the box. Whiskers represent the lowest and highest observations within 1.5 times the first and third quartile.

First, we assessed hβD1 (*DEFB1*) expression in the human stomach in *H. pylori* infected and uninfected patients, in comparison with hβD2 (*DEFB4A*) expression. *DEFB1* mRNA expression levels were threefold lower in gastric biopsies from 31 *H. pylori* infected compared with 23 uninfected patients (*P* = 0.005; Fig. 1A). In agreement with previous studies (Wada *et al*., 1999; Hamanaka *et al*., 2001; Uehara *et al*., 2003; Boughan *et al*., 2006; Bauer *et al*., 2013), *DEFB4A* expression levels were elevated in *H. pylori* infected gastric biopsies (*P* = 0.001; Fig. 1A). Median *DEFB1* expression was twofold lower with *cagA*+ strain infections compared with *cagA*− infections, while *DEFB4A* expression was significantly higher (*P* = 0.028 and *P* = 0.006 respectively; Fig. 1A). In a manner similar to other studies on gastric mucosal defensins, to determine differences in protein expression, gastric biopsies were lysed and the concentrations of hβD1 and hβD2 were quantified by ELISA (Bauer *et al*., 2013). As found by RT-qPCR, hβD1 concentrations were significantly lower in biopsies from 10 infected patients compared with five uninfected patients (*P* = 0.001; Fig. 1B), while hβD2 protein concentrations were higher (*P* = 0.001). Lower hβD1 and higher hβD2 concentrations were also detected in the presence of a *cagA*+ infection (*P* = 0.016 and *P* = 0.004 respectively; Fig. 1B).

Next, we examined associations of *DEFB1* and *DEFB4A* expression with the intensity of inflammation as assessed by histopathology, scoring gastric antral tissue sections from the *H. pylori* infected patients. Sixfold lower *DEFB1* mRNA levels were observed in samples with grade 3 inflammation compared with those with grade 1 (*P* = 0.045; Fig. 1C). There was an opposing trend but no significant differences in *DEFB4A* expression. Finally, we investigated the relationship between hβD1 and *H. pylori* colonization density *in vivo*, also by histopathology. A twofold lower *DEFB1* mRNA level was observed in samples with grade 3 density compared with those with grade 1 (*P* = 0.009; Fig. 1D), suggesting a link between its expression and control of bacterial density. Again, no significant differences were observed for *DEFB4A* expression.

### hβD1 is downregulated in epithelial cells by pathogenic strains of *H. pylori in vitro*

**Figure 2:**
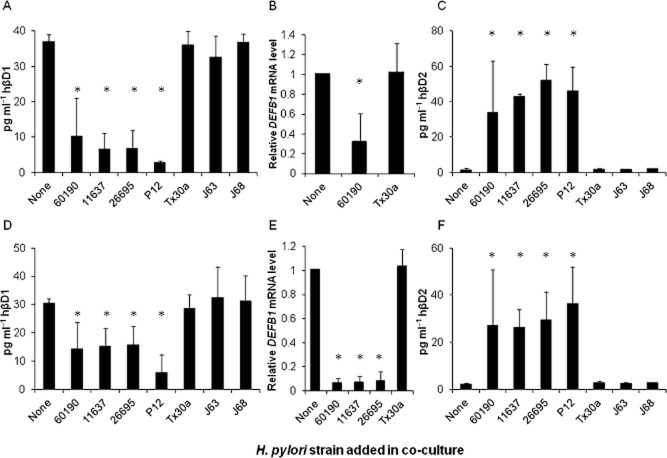
Analysis of hβD1 expression during *H. pylori* infection *in vitro*. MKN7 (A–C) and AGS (D–F) cell lines were infected with *H. pylori* strains 60190, 11637, 26695, P12 (all *cagA* positive and expressing the s1/m1 form of *vacA*), and Tx30a, J63, J68 (*cag*PAI−, *vacA* s2/m2) for 24 h. hβD1 and hβD2 protein concentrations in culture supernatants were measured by ELISA (A, C, D and F). hβD1 mRNA expression was measured by RT-qPCR (B and E), and data presented as fold differences relative to that measured in uninfected cells. Bars depict mean expression levels from three independent experiments and error bars show standard deviations. The asterisk (*) indicates a significant difference in expression compared with uninfected cells (*P* < 0.01).

To assess hβD1 expression by epithelial cells in response to *H. pylori* infection *in vitro*, we co-cultured the MKN7 human gastric epithelial cell line [reported to have the most similar characteristics to normal human gastric mucosal cells (Linden *et al*., 2007)] for 24 h with the *cag*PAI+ *vacA* s1/m1 *H. pylori* strains 60190, 26695, 11637 and P12, and the *cag*PAI− *vacA* s2/m2 strains Tx30a, J63 and J68 at a multiplicity of infection (moi) of 100 bacteria per cell. ELISA assays showed that mean hβD1 protein concentrations in culture supernatants were consistently > 73% lower following infection with the *cag*PAI+ strains compared with uninfected cells (*P* < 0.001 for each; Fig. 2A), but no effects were induced by any of the *cag*PAI− strains. This result was confirmed for 60190 and Tx30a strains by RT-qPCR (Fig. 2B). Conversely, in the same experiment the *cag*PAI+ strains induced marked increases in hβD2 release (*P* < 0.01 for all; Fig. 2C) as previously reported (Wada *et al*., 1999; O'Neil *et al*., 2000; Uehara *et al*., 2003). To demonstrate that the findings were not a cell line-specific anomaly, we also conducted experiments with AGS cells in parallel and obtained similar results, although lower concentrations of defensins were detected (Fig. 2D–F). These data show that pathogenic *H. pylori* strains potently downregulate hβD1 expression by different gastric epithelial cell lines.

### The *H. pylori* *cag*PAI induces hβD1 downregulation

**Figure 3:**
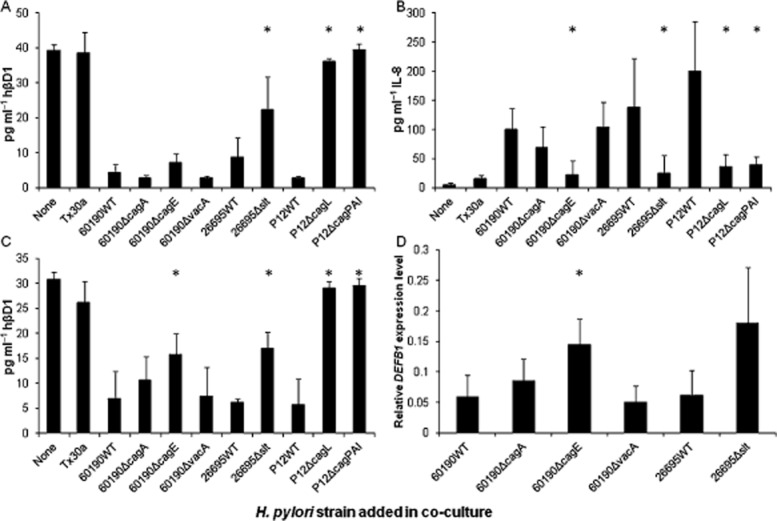
The effect of bacterial virulence factors on hβD1 expression *in vitro*. MKN7 (A and B) and AGS cells (C and D) were infected with *H. pylori* strains Tx30a, 60190WT, 60190Δ*cagA*, 60190Δ*cagE*, 60190Δ*vacA*, 26695WT, 26695Δ*slt*, P12WT, P12Δ*cagL* and P12Δ*cag*PAI (moi = 100). hβD1 (A and C) and IL-8 (B) concentrations in culture supernatants were measured by ELISA after 24 h. The asterisk (*) indicates a significant difference in concentration when comparing effects of mutant strains to their parental strain 60190WT, 26695WT or P12WT (*P* < 0.01). Fold differences in hβD1 mRNA expression relative to uninfected cells were quantified by RT-qPCR (D). The asterisk (*) indicates a significant difference in expression level compared with 60190WT-infected cells (*P* < 0.05). Bars represent the mean of three independent experiments and error bars show standard deviations.

As we observed hβD1 downregulation *in vitro* only when cells were infected with *cag*PAI+ *vacA* s1/m1 *H. pylori* strains, we next aimed to determine which bacterial genes influenced the expression of hβD1. To achieve this, hβD1 protein and mRNA expression levels were assessed when MKN7 or AGS cells were co-cultured with the wild-type strain 60190 (60190WT), or its isogenic mutants 60190Δ*cagE* (which does not express the *cag*PAI-encoded T4SS), 60190Δ*cagA* (which expresses the T4SS but does not translocate CagA into host cells) and a *vacA* null mutant (60190Δ*vacA*). The reduction in hβD1 in MKN7 and AGS cells was less marked for the 60190Δ*cagE* mutant than 60190WT (significant difference in AGS cells only, *P* = 0.01) indicating that the *cag*PAI contributed to hβD1 downregulation. However, the 60190Δ*cagA* strain downregulated hβD1 by a similar extent to the wild-type strain, for both mRNA and protein levels, showing that the injected T4SS effector protein CagA was not involved in this process (Fig. 3A, C and D). We also found no difference in hβD1 expression from co-culture of epithelial cell lines with the 60190Δ*vacA* mutant (Fig. 3A, C and D). As a control for the performance of the mutants in the assays, IL-8 concentrations were also measured. Effects of all mutants were in line with previous reports (Viala *et al*., 2004; Argent *et al*., 2008; Gorrell *et al*., 2013) (Fig. 3B).

As *H. pylori* peptidoglycan processed by the lytic transgycosylase Slt has also been reported to be translocated into epithelial cells via the *cag*PAI-encoded T4SS, inducing activation of NOD1, NF-κB signalling and secretion of the pro-inflammatory cytokine IL-8 (Viala *et al*., 2004), we investigated whether this process contributed to hβD1 downregulation. Cells were cultured with an *slt* (HP0645) null mutant derived from *H. pylori* strain 26695 (26695Δ*slt*) (Viala *et al*., 2004; Chaput *et al*., 2007). This mutant generates up to 40% less cell wall disaccharide tripeptide than the wild-type (26695WT) but has comparable growth rates with the wild-type strain and has no defects in the formation of the T4SS. We showed that the 26695Δ*slt* strain induced significantly less hβD1 downregulation compared with 26695WT in MKN7 and AGS cells (*P* = 0.01) but this did not completely reverse the effect (Fig. 3A and C).

Finally we co-cultured cells with a complete *cag*PAI null mutant derived from the P12 strain (P12Δ*cag*PAI), and confirmed that levels of hβD1 expression were similar to that observed in uninfected cells. Similarly a *cagL* null mutant (P12Δ*cag*L), in which the T4SS is incapable of interacting with epithelial cells via α_5_β_1_ integrin, did not downregulate hβD1 expression. These results show that the *cag*PAI induces hβD1 downregulation, possibly through CagL-α_5_β_1_ integrin interactions and delivery of cell wall disaccharide tripeptides, rather than via delivery of CagA.

### *H. pylori* usurps NF-κB signalling to downregulate hβD1

**Figure 4:**
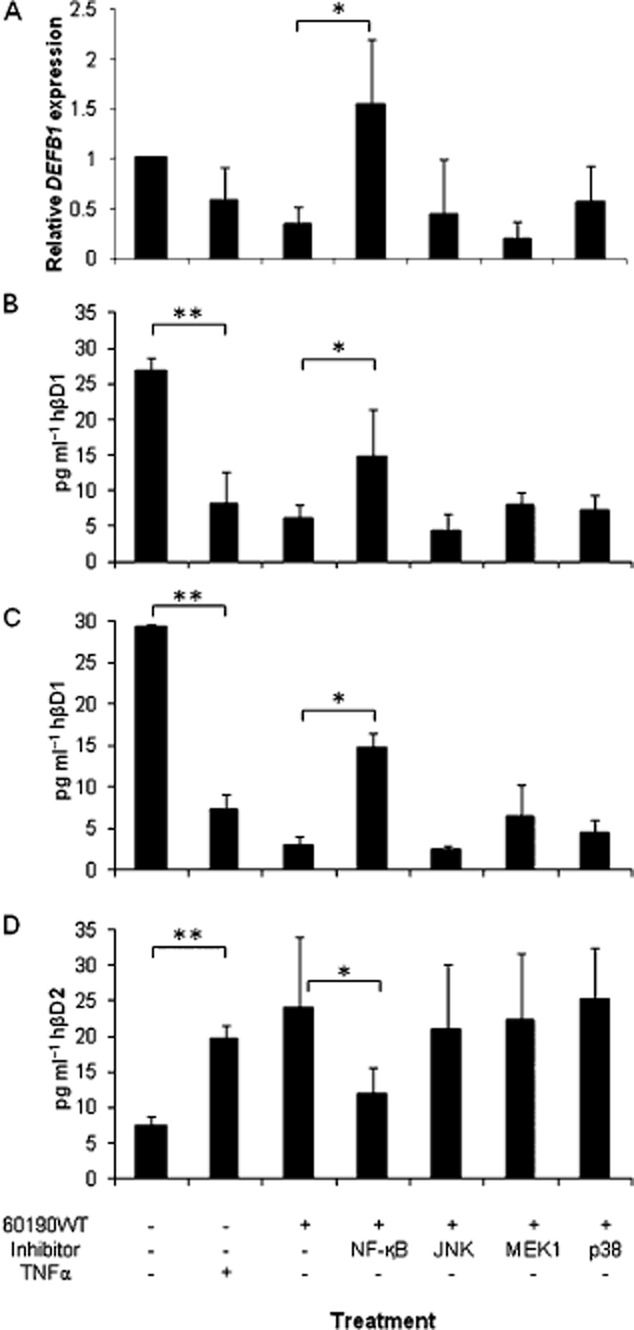
Assessing the signalling pathways involved in modulation of hβD1 expression *in vitro*, using inhibitor drugs. Expression of hβD1 mRNA (A) and protein (B and C), and also hβD2 protein (D) was assessed after treating 60190WT *H. pylori* infected AGS (A and B) and MKN7 (C and D) cells with 6-amino-4-(4-phenoxyphenylethylamino)quinazoline (NF-κB activation inhibitor), SP600125 (JNK inhibitor), U0126 (MEK1 inhibitor), or SB203586 (p38 inhibitor), prior to and during incubation. Treatment with the drug diluent alone was included as a negative control. TNFα treatment was included as a positive control inducer of NF-κB activation. mRNA expression levels are given as a fold difference relative to uninfected and untreated cells. *hβD1 significantly higher and hβD2 lower in NF-κB inhibitor-treated cells, compared with controls (*P* < 0.05). **hβD1 significantly lower and hβD2 higher in TNFα-treated, compared with untreated cells (*P* < 0.05). Bars represent the mean from three independent experiments and error bars show standard deviations.

We next aimed to determine the intracellular signalling pathways through which *H. pylori* regulates hβD1 expression. Sequence analysis of the *DEFB1* gene identified binding sites in the promoter for NF-κB1 (p50 subunit of NF-κB) and Activator Protein (AP)-1 (Prado-Montes de Oca, 2010), which implies regulation of hβD1 transcription through NF-κB and/or MAP kinase signalling. Given the observed association between the *cag*PAI and hβD1 expression, we investigated the role of NF-κB and the individual ERK, p38 and JNK MAP kinase signalling pathways in hβD1 downregulation during infection. AGS and MKN7 cells were cultured with *H. pylori* strain 60190WT in the presence of specific drug inhibitors of each pathway respectively. Effects on *DEFB1* mRNA, and hβD1 and hβD2 protein were examined (Fig. 4). Uninfected cells were treated with recombinant TNFα as a positive control for activation of NF-κB, and this reduced *DEFB1* expression, reduced hβD1 secretion (*P* = 0.001), and increased hβD2 release compared with untreated cells (*P* = 0.01). As previously, the 60190WT strain reduced hβD1 and increased hβD2 expression. The ERK, p38 and JNK kinase inhibitors had a slight but no significant impact on *H. pylori*-induced hβD1 downregulation. In contrast, the NF-κB inhibitor blocked these effects significantly (two- to fourfold difference in hβD1 concentrations between cultures infected with 60190WT in the presence and absence of NF-κB inhibitor; *P* = 0.05 and *P* = 0.01 in AGS and MKN7 cells respectively; Fig. 4B and C). These results confirm the importance of the NF-κB signalling pathway in *H. pylori*-modulated expression of hβD1 expression.

**Figure 5:**
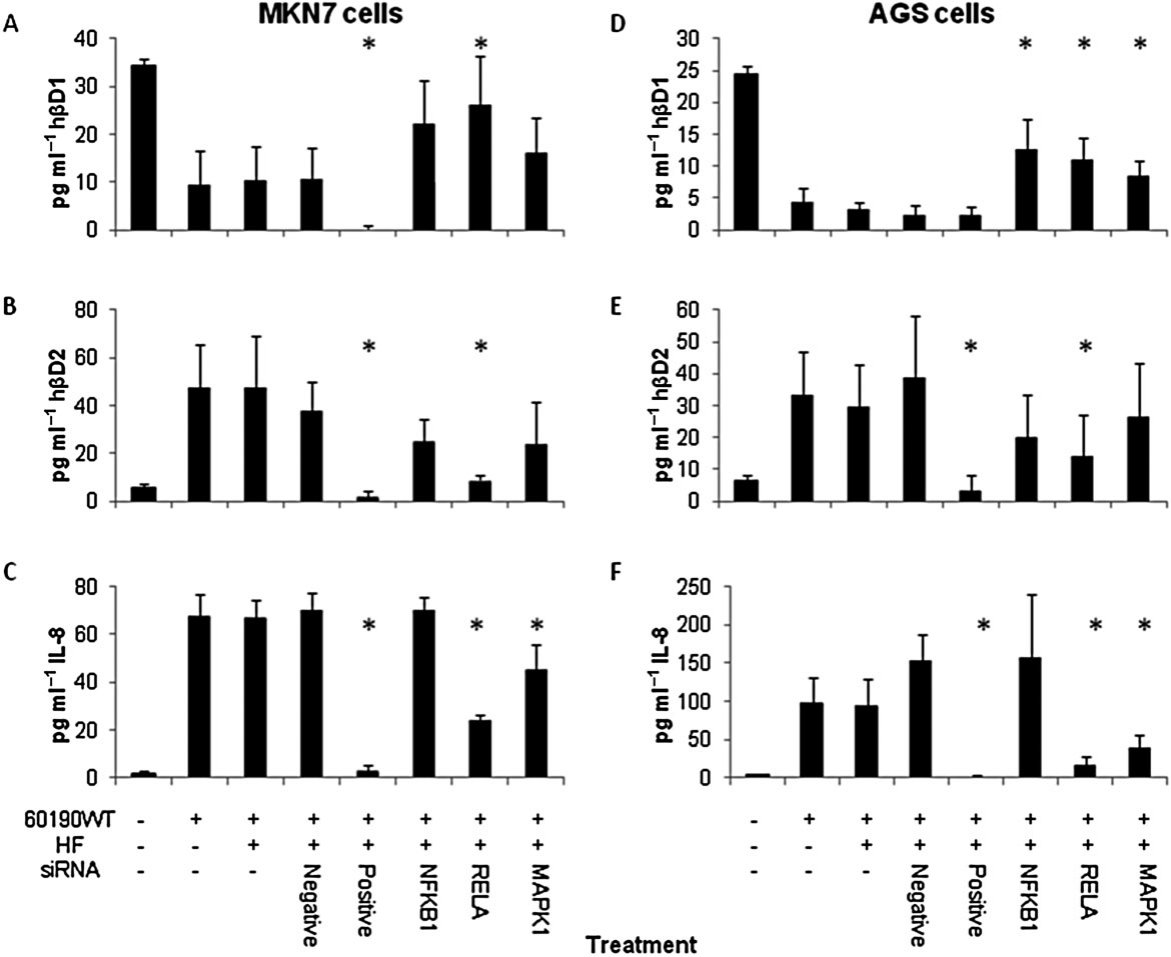
Assessing the signalling pathways involved in modulation of hβD1 expression *in vitro*, using gene silencing. hβD1 (A and D), hβD2 (B and E) and IL-8 (C and F) concentrations 24 h after infecting MKN7 (A–C) and AGS (D–F) cells with 60190WT *H. pylori*. Cells were pre-treated 48 h previously with siRNA duplexes in HiPerfect transfection reagent (HF). siRNA treatments targeted the *NFKB1* (NFκBp50), *RELA* (NFκBp65) and *MAPK1* genes. Negative control duplexes were non-silencing, whereas positive control duplexes targeted genes necessary for cell survival. *Significantly different concentration compared with cells treated with negative control siRNA (*P* < 0.05). Bars represent the mean from three independent experiments and error bars show standard deviations.

To confirm the data and investigate the mechanisms further, small interference RNA (siRNA) experiments were performed to silence expression of *NFKB1* (which encodes the NF-κBp50 subunit), and *RELA* (NF-κBp65 subunit). *MAPK1* siRNA duplexes were also tested since the MAP kinase pathway is known to be stimulated by *cagA*-independent *cag*PAI signalling. Western blots confirmed the gene knock-downs (Fig. S1). 60190WT-infected cells previously treated with *NFKB1* or *RELA* siRNA expressed two- to fivefold higher concentrations of hβD1 compared with those treated with negative control duplexes (*P* < 0.05 for both siRNAs in MKN7 and AGS cells; Fig. 5A and D). hβD2 expression in *H. pylori-*infected MKN45 cells is reportedly controlled by the p65 homodimeric form of NF-κB (Wada *et al*., 2001). Threefold lower concentrations of hβD2 were detected following *RELA* silencing in both cell lines (*P* < 0.05); effects of *NFKB1* siRNA were less marked (Fig. 5B and E). *RELA* silencing also had a dramatic effect on IL-8 responses, but *NFKB1* siRNA had no effect (Fig. 5C and F). *MAPK1* siRNA treatment also had an effect on *H. pylori-*induced hβD1 expression, with significantly increased concentrations in AGS cell supernatants (*P* = 0.05). These data confirm the importance of NF-κB in the *H. pylori-*mediated downregulation of hβD1 expression and upregulation of hβD2 expression. They also indicate some involvement of the ERK pathway.

## Discussion

Antimicrobial peptides play a vital role during infection, acting as a key line of defence against invading microbes and also as essential components in modulating the immune response to infections. While expression of hβD2 and hβD4 is inducible and upregulated in response to *H. pylori* infection, hβD1 is normally constitutively expressed by epithelial cells in the absence of *H. pylori*. Mice with a deletion in the homologous *mBD1* gene have an impaired capacity to combat bacterial infections (Morrison *et al*., 2002; Moser *et al*., 2002), reflecting the importance of this AMP as a component of the innate anti-bacterial immune response. However, there is conflict in the literature concerning how hβD1 is differentially expressed during *H. pylori* infection (Bajaj-Elliott *et al*., 2002; Taha *et al*., 2005; Kocsis *et al*., 2009; Vordenbaumen *et al*., 2010). In agreement with the study by Taha *et al*., we found that mRNA expression of hβD1 was downregulated in the *H. pylori*-infected human gastric mucosa and also in infected gastric epithelial cells *in vitro*. Two studies reporting upregulated hβD1 expression in infected epithelial cell lines *in vitro* used the same primer sequences (Bajaj-Elliott *et al*., 2002; Kocsis *et al*., 2009). When we performed additional tests using these however, the trends in our data remained the same, i.e. hβD1 expression was downregulated by infection by functional T4SS *cag*PAI+ *H. pylori* (data not shown). We were also able to confirm our findings using ELISA to quantify hβD1 protein both in gastric biopsy tissue and in culture supernatants, which validates our mRNA data.

Our data show that hβD1 expression is modulated during *H. pylori* infection. Downregulation of hβD1 expression has previously been observed in the intestinal mucosa of patients infected with *Shigella dysenteriae* (Islam *et al*., 2001), or those with Crohn's disease or ulcerative colitis (Wehkamp *et al*., 2003). There is also a precedent for hβD1 downregulation in epithelial cells *in vitro*. Culturing intestinal epithelial cells with the enteric pathogens *Vibrio cholerae*, enterotoxigenic *Escherichia coli* and *S. dysenteriae* suppressed hβD1 expression in a manner involving protein kinase A and ERK MAP kinase signalling (Chakraborty *et al*., 2008). Infections of airway and gingival epithelial cells with influenza virus, Herpes simplex virus 1 and Sendai virus was also recently reported to downregulate hβD1 expression. This process required live virus, but the mechanism remains unknown (Ryan *et al*., 2011).

We found that *cag*PAI+ wild-type strains markedly suppressed hβD1 expression, while three *cag*PAI− strains consistently did not. Analysis of bacterial factors demonstrated that hβD1 downregulation was *cagA* independent. Although our gastric biopsy data showed lower hβD1 expression in those infected with *cagA*+ strains, we have merely used this as a marker for presence of the *cag*PAI. *In vitro*, hβD1 downregulation was completely abrogated in cells infected with *cag*PAI- or *cagL-*deficient mutants, and partially reversed with the *slt* mutant. This indicated that the suppression was mediated by T4SS engagement of the α_5_β_1_ integrin and NOD1 activation in epithelial cells. We then investigated NF-κB- and MAPK-dependent downregulation of hβD1, given the known action of *cag*PAI-containing strains upon these signalling pathways (Brandt *et al*., 2005). Interestingly, increased hβD1 expression was observed when NF-κB signalling was inhibited, and was reduced with TNFα-mediated NF-κB activation. NF-κB response elements have been described in the *DEFB1* promoter sequence (Prado-Montes de Oca *et al*., 2009). The role of *H. pylori* induced NF-κB signalling in the suppression rather than induction of gene expression is somewhat unusual, but not unknown. For example, suppression of H,K-ATPase expression, the enzyme mediating gastric acid secretion, was observed in *H. pylori* infected AGS cells and found to involve T4SS-dependent, CagA-independent NF-κB activation (Saha *et al*., 2008; 2010).

The NF-κB family of transcription factors consists of five members, and NF-κB exists as a homo- or heterodimer of these subunits. Of these, p50 and p52 lack the transcription activation domain necessary for transcription. Binding of these homodimers to a promoter can block transcription of the target gene (Hayden and Ghosh, 2008). Saha *et al*. showed that infection of AGS cells with a *cag*PAI+ strain of *H. pylori* induced transfer of both homodimeric p50/p50 and heterodimeric p65/p50 forms to the nucleus. Expression of H,K-ATPase was repressed by the binding of p50/p50 NF-κB to the HKα promoter (Saha *et al*., 2008). The *DEFB1* gene promoter is known to have a p50-binding domain, therefore p50 homodimers or p65/p50 heterodimers could potentially bind (Prado-Montes de Oca *et al*., 2009). We found that silencing of *NFBK1* and *RELA* equivalently prevented the inhibition of hβD1 expression, therefore each of these genes plays a role and the inhibitory effect of p50 homodimers appears a less likely explanation. Another possibility is that NF-κB activation (p65/p50) stimulates expression of host factors which then block hβD1 gene expression, for example olfactomedin 4, which inhibits NF-κB activation in a feedback mechanism involving NOD1 (Liu *et al*., 2010a), and various microRNAs (Xiao *et al*., 2009; Tang *et al*., 2010; Liu *et al*., 2010b). Our finding that hβD1 suppression could be induced by TNFα, which is known to stimulate activation and nuclear translocation of NF-κBp65 in AGS cells (Robinson *et al*., 2008), is novel and adds weight to this theory. TNFα could also be exerting an effect on defensin expression in the stomach, and it would be interesting to test this using animal models. Incubation of other types of epithelial cells with NF-κB inhibitors or TNFα has not been shown to influence hβD1 expression (Zhao *et al*., 1996; O'Neil *et al*., 1999; Joly *et al*., 2005); however, defensin responses are known to be cell line dependent (Grubman *et al*., 2010).

As a further control for our experiments, we measured expression of the more widely studied defensin hβD2. In accordance with others, we found this to be increased in response to *H. pylori* both *in vivo* and *in vitro* (Wada *et al*., 1999; Boughan *et al*., 2006; Bauer *et al*., 2013), and increased further with *cag*PAI+ strains (Hornsby *et al*., 2008; Grubman *et al*., 2010). Bauer *et al*. found that although *DEFB4* mRNA was elevated in the infected gastric mucosa, this trend could not be shown with protein concentrations (Bauer *et al*., 2013). The defensin concentrations detected in our study were lower, possibly because we used a buffer with a lower detergent content when preparing the lysates (Staples *et al*., 2013). This possible explanation for the discrepant results between the studies warrants further investigation. Our mechanistic data on hβD2 agreed with that of Grubman *et al*., who found that NOD1 activation induced by *cag*PAI+ strains induced *DEFB4* mRNA expression in AGS cells. Interestingly they showed that *DEFB4* expression could also be induced in HEK293 cells by stimulation with TNFα (Grubman *et al*., 2010). We found similar trends to our *in vivo* data using two different cell lines, and also confirmed the findings of others. This is very encouraging, but further studies are needed with a wider range of cell types, and using other methods, e.g. luciferase reporter assays of *DEFB1* and *DEFB4* gene promoter activity, and immunohistochemistry analysis of biopsy tissues. Using a defensin ELISA on whole biopsy lysates does not take account of the possibility that increased inflammatory cells in infected tissue influenced the findings, which were normalized for total protein content. The range of biopsy protein concentrations among the groups, however, were similar.

We have shown that epithelial cell hβD1 expression is downregulated during *H. pylori* infection, but the importance of such modulation is still not completely clear. It has recently come to light that hβD3 expression is also suppressed during prolonged *H. pylori* infection of AGS cells via a CagA-dependent mechanism, and that its expression *in vivo* is also reduced in gastric biopsies from infected patients (Bauer *et al*., 2012; 2013). The fact that high colonization densities *in vivo* correspond with lower hβD1 expression indicates that reducing the level of hβD1 may contribute to the persistence of the bacterium in the gastric mucosa, but a role for hβD3 suppression is also likely to be important. Additionally, hβD1 bactericidal activity has been reported to be synergistic with hβD2 and the cathelicidin LL-37 (George *et al*., 2003; Hase *et al*., 2003), both of which have bactericidal activity against the bacterium. Therefore, downregulation of hβD1 may also limit the consequences of hβD2 and LL-37 activity, providing an additional benefit over merely reducing hβD1 expression.

In conclusion, we have demonstrated an NF-κB-dependent downregulation of hβD1 expression during *H. pylori* infection, which was dependent on CagA-independent *cag*PAI signalling. In agreement with the *in vitro* experiments, lower-level expression of hβD1 in the infected human gastric mucosa was significantly associated with *cag*PAI+ strains, more severe inflammation and higher colonization densities. We suggest that *H. pylori-*induced modulation of hβD1 expression may contribute to the persistence of *H. pylori* in the gastric mucosa.

## Experimental procedures

### Tissue samples

Antral gastric biopsies were donated by 31 *H. pylori*-infected and 23 uninfected patients attending the University Hospital, Nottingham, for routine upper gastrointestinal endoscopy, with informed written consent and approval from the Nottingham Research Ethics Committee. *H. pylori* status was determined by rapid urease test, bacterial culture and histology. Samples were not collected from patients taking proton pump inhibitors, non-steroidal anti-inflammatory drugs, or antibiotics in the 2 weeks preceding endoscopy. Bacterial isolates were PCR-genotyped for *cagA* status as previously described (Hussein *et al*., 2008). Biopsy specimens for histology were formalin-fixed, paraffin-embedded, cut to 4 μm thickness, and stained with haematoxylin and eosin or toluidine blue for assessment of inflammation and *H. pylori* colonization density respectively. Grading was carried out using the modified Sydney Scoring System (0 = not present, 1 = mild, 2 = moderate and 3 = substantial) by an experienced histopathologist (AMZ) who was blinded to other data (Genta and Dixon, 1995). Biopsies for RNA analysis were immediately preserved in RNAlater (Sigma-Aldrich, UK).

### Gastric biopsy lysates

Gastric biopsies from five uninfected and 10 infected patients (five with *cagA*+ strains) were homogenized according to a previously described method (Staples *et al*., 2013). Single biopsies were suspended in 300 μl PBS containing 2 mM Mg^2+^ (Sigma), 25 U ml^−1^ Benzonase® nuclease (Novagen, Germany), and protease inhibitors (complete mini [EDTA-free], Roche, Germany), processed on ice using disposable pestles and filter tips. Samples were clarified by centrifugation at 10 000 *g* for 10 min at 4°C. Supernatants were aliquoted into LoBind tubes (Eppendorf), tested for total protein concentration using a bicinchoninic acid (BCA) assay kit (Pierce, IL, USA), and stored at −80°C. Supernatants from infected and uninfected donors contained similar protein concentrations (medians 1.77 and 1.54 mg ml^−1^ respectively).

### Cell lines and bacterial strains

The human gastric epithelial MKN7 cell line (kind gift from Dr Sara Linden, University of Gothenburg, Sweden) was maintained in RPMI1640 medium supplemented with 10% heat-inactivated fetal bovine serum (FBS) (Sigma-Aldrich). AGS cells (ATCC CRL-1739™) were grown in nutrient mixture F12 Ham supplemented with 10% FBS and 2 mM l-glutamine (Sigma-Aldrich). All cell lines were incubated at 37°C in a 5% CO_2_ humidified atmosphere. *cag*PAI+ *H. pylori* strains 60190, 11637, 26695, P12 and *cag*PAI− isolates Tx30a, J63 and J68 (Boughan *et al*., 2006; Corcoran *et al*., 2007; Keates *et al*., 2007) were cultured on Blood agar base 2 containing 5% (v/v) horse blood (Oxoid, Cambridge, UK) at 37°C under microaerobic conditions (Argent *et al*., 2004). Isogenic mutants deficient in *vacA* (60190Δ*vacA*), *cagA* (60190Δ*cagA*) and *cagE* (60190Δ*cagE*) derived from the 60190 strain (Argent *et al*., 2008), *cag*PAI- and *cagL-*deficient mutants (P12Δ*cag*PAI and P12Δ*cagL*) derived from the P12 strain (Kwok *et al*., 2007), and an *slt* deletion mutant (26695Δ*slt*) derived from the 26695 strain [kindly donated by Dr Richard Ferrero, Monash University, Victoria, Australia (Viala *et al*., 2004)], were also used.

### *In vitro* culture experiments

Using methods based on those of Bajaj-Elliott *et al*. (2002), 5 × 10^4^ MKN7 or AGS cells per well were seeded in 24-well culture plates with the appropriate medium and allowed to adhere at 37°C in a 5% CO_2_ air-humidified atmosphere for 24 h. The medium was replaced with a suspension of *H. pylori* at a multiplicity of infection of 100 bacteria per epithelial cell, and cultures were incubated for a further 24 h. Multiplicities of infection were confirmed by viable counting. For quantification of defensins and IL-8 concentrations in supernatants, 1 × 10^5^ epithelial cells per well were seeded, and co-cultures were carried out using serum-free F12 medium.

### Defensin and IL-8 ELISA assays

After co-culture of epithelial cells with *H. pylori*, supernatants were aliquoted, frozen at −80°C and thawed once only. Biopsy lysates were thawed and tested immediately for defensins. hβD1 and hβD2 assays were performed using Human BD-1 and BD-2 ELISA Development Kits (PeproTech, UK) and IL-8 concentrations were determined with a Human IL-8 CytoSet™ ELISA (Invitrogen), according to manufacturers' instructions and with a standard curve on each plate. Typical sensitivity limits (mean plus 3 standard deviations of six replicate 0 pg ml^−1^ control wells) were 0.5 pg ml^−1^ hβD1, 4.5 pg ml^−1^ hβD2 and 5.1 pg ml^−1^ IL-8.

### Reverse transcriptase PCR (RT-qPCR)

RNA was extracted from antral gastric biopsies and cell lines using an RNeasy Mini kit (QIAGEN, Crawley, UK) according to the manufacturer's instructions. cDNA was generated from 100 ng RNA using Superscript reverse transcriptase II, with oligo (dT) primers (Invitrogen). Real-time PCR was performed using the Rotor-Gene 3000 real-time PCR system (QIAGEN). First stage RT-PCR samples, produced in the absence of reverse transcriptase from each RNA sample, were tested in parallel to detect genomic DNA contamination. Samples were run in duplicate and the results were analysed using the Pfaffl method (Pfaffl, 2001). Relative gene expression levels were determined by normalizing against human glyceraldehyde 3-phosphate dehydrogenase (*GAPDH*) mRNA levels, and data were presented as a fold difference in comparison with an uninfected reference sample. For assessing expression *in vivo*, the uninfected comparator consisted of cDNA synthesized from pooled purified RNA extracted from biopsies of 10 randomly selected *H. pylori*-negative patients. For *in vitro* analysis, RNA was purified from epithelial cells cultured under different conditions for 24 h. The uninfected negative controls in each experiment were taken as the negative comparator. A commercial human cDNA standard (BD Biosciences; Oxford, UK) was included as a positive control in all assays.

Quantification of hβD1 mRNA was carried out using a QuantiTECT™ SYBR Green PCR kit with commercial primers (QIAGEN). Amplification of hβD2 was carried out over 45 cycles of 15 s at 95°C, 30 s at 61°C and 30 s at 72°C (Primer sequences: hβD2 forward: 5′-CTGATGCCTCTTCCAGGTGTTT-3′; hβD2 reverse: 5′-GAGACCACAGGTGCCAATTTG-3′; *GAPDH* forward: 5′-CCACATCGCTCAGACACCAT-3′; *GAPDH* reverse: 5′-GGCAACAATATCCACTTTACCAGAGT-3′). No-template controls were included in each run.

### Inhibitor studies

Epithelial cells were pre-treated with specific chemical inhibitors (Merck, Nottingham, UK) for 60 min prior to and during bacterial stimulation. The drugs used were U0126 (10 μM; MEK 1 inhibitor), SP600125 (10 μM; JNK inhibitor), SB203586 (10 μM; p38 inhibitor) and 6-amino-4-(4-phenoxyphenylethylamino)quinazoline (1 μM; NF-κB activation inhibitor). Cultures were incubated as described above and defensin expression levels were assessed. As a positive control inducer of NF-κB activation (Robinson *et al*., 2008), cells were treated with 50 ng ml^−1^ recombinant TNFα (PeproTech).

### siRNA transfections

Validated siRNA duplexes targeting *NFKB1*, *RELA* and *MAPK1* mRNA (QIAGEN) were prepared according to the manufacturer's instructions. Non-silencing AllStars Hs Negative Control siRNA and AllStars Hs Cell Death Control siRNA (positive control) (QIAGEN) were tested in parallel. Epithelial cells were seeded at 1 × 10^5^ per well in 24-well plates and treated with 10 nM siRNA suspended in HiPerfect transfection regent (QIAGEN). Controls were treated with HiPerfect only, or PBS. The cells were incubated for 48 h at 37°C in 5% CO_2_, when a high degree of cell death was observed in the positive control wells. This siRNA construct targets genes that are indispensable for cell survival, thus cell death confirmed successful transfection. *NFKB1, RELA* and *MAPK1* gene knock-down was confirmed by Western blotting (Fig. S1) using rabbit antibodies against NF-κB p50 (Cell Signaling Technology, MA, USA), NF-κB p65 (Millipore, MA, USA), MAPK1/ERK (Source BioScience UK) and actin (Sigma-Aldrich), with an anti-rabbit IgG-peroxidase conjugate (Sigma-Aldrich) and chemiluminescent ECL substrate (GE Healthcare, UK). Medium was removed from the wells before infecting with *H. pylori* for a further 24 h.

### Statistical analysis

Statistical analyses were carried out using GraphPad Prism 6 software. A *P* value ≤ 0.05 was taken as indicative of a significant difference. *In vivo* data were displayed in box-and-whisker plots, and compared using a Mann–Whitney *U* test or, for multiple parameters, Kruskal–Wallis tests with a *post hoc* Dunn's multiple comparison. *In vitro* data were described using means and standard deviations, and comparisons between groups were made using one-way anova with a Dunnett's *post hoc* test for multiple variates.

## Supporting information

Fig S1 Confirmation of gene silencing in AGS cells treated with siRNA duplexes by Western blotting. Cells seeded in 24-well plates were transfected with siRNA to target expression of NFKB1, RELA, and MAPK1. Non-silencing negative control duplexes (Neg) were also used. After 48 h, cells were harvested into SDS-PAGE sample buffer. Western blots were probed, stripped and re-probed using antibodies against NF-κB p50 (NFKB1 gene product), NF-κB p65 (RELA gene product), MAPK1/ERK and beta actin.
